# Mycosynthesis of Metal-Containing Nanoparticles—Synthesis by Ascomycetes and Basidiomycetes and Their Application

**DOI:** 10.3390/ijms24010304

**Published:** 2022-12-24

**Authors:** Martin Šebesta, Hana Vojtková, Veronika Cyprichová, Avinash P. Ingle, Martin Urík, Marek Kolenčík

**Affiliations:** 1 Institute of Laboratory Research on Geomaterials, Faculty of Natural Sciences, Comenius University in Bratislava, Ilkovičova 6, 841 04 Bratislava, Slovakia; 2 Department of Environmental Engineering, Faculty of Mining and Geology, VŠB−Technical University of Ostrava, 17. listopadu 2172/15, 708 00 Ostrava, Czech Republic; 3 Biotechnology Centre, Department of Agricultural Botany, Dr. Panjabrao Deshmukh Agricultural University, Akola 444 104, India; 4 Department of Soil Science and Geology, Institute of Agronomic Sciences, Faculty of Agrobiology and Food Resources, Slovak University of Agriculture in Nitra, Tr. A. Hlinku 2, 949 76 Nitra, Slovakia

**Keywords:** nanofertilizer, fungal synthesis, antimicrobial agent, catalyst, biomedicine, nanobiosensors, precision agriculture, extracellular extracts, intracellular extracts

## Abstract

Fungi contain species with a plethora of ways of adapting to life in nature. Consequently, they produce large amounts of diverse biomolecules that can be generated on a large scale and in an affordable manner. This makes fungi an attractive alternative for many biotechnological processes. Ascomycetes and basidiomycetes are the most commonly used fungi for synthesis of metal-containing nanoparticles (NPs). The advantages of NPs created by fungi include the use of non-toxic fungus-produced biochemicals, energy efficiency, ambient temperature, pressure conditions, and the ability to control and tune the crystallinity, shape, and size of the NPs. Furthermore, the presence of biomolecules might serve a dual function as agents in NP formation and also capping that can tailor the (bio)activity of subsequent NPs. This review summarizes and reviews the synthesis of different metal, metal oxide, metal sulfide, and other metal-based NPs mediated by reactive media derived from various species. The phyla ascomycetes and basidiomycetes are presented separately. Moreover, the practical application of NP mycosynthesis, particularly in the fields of biomedicine, catalysis, biosensing, mosquito control, and precision agriculture as nanofertilizers and nanopesticides, has been studied so far. Finally, an outlook is provided, and future recommendations are proposed with an emphasis on the areas where mycosynthesized NPs have greater potential than NPs synthesized using physicochemical approaches. A deeper investigation of the mechanisms of NP formation in fungi-based media is needed, as is a focus on the transfer of NP mycosynthesis from the laboratory to large-scale production and application.

## 1. Introduction

In the last few decades, research on the development of alternative nanoparticle (NP) production has attracted a great deal of attention due to the plethora of novel applications of NPs in various industries. NPs are solid particles with a size range between 1 and 100 nm in at least one dimension. Due to their small size, they have a large surface area that provides them with novel properties [1]. Metal-containing NPs were shown to have antimicrobial and anti-cancer properties, catalytic and photocatalytic activity, and interesting optical and magnetic properties [2]. These properties of NPs are size specific since with a decrease in size there is an increase in the active surface area of the NPs. Hence, the antimicrobial and other properties of NPs often increase with a decrease in NP size [3]. However, this is not true for all applications. Kusiak-Nejman et al. [4] found in their experiment that when ZnO NPs of sizes 23, 27, 32, 44, 57, and 71 nm were applied to photocatalytic degradation of phenol in a water solution, the largest 71 nm-sized ZnO NPs had the highest photocatalytic activity, and the increase in size correlated with increased photocatalytic activity. Thus, even though there is a general rule of higher activity in smaller NPs, for each application, the most suitable size of NPs needs to be evaluated depending on the required properties that can be fine-tuned with NP size.

Metal-containing NPs can be produced via “bottom-up” or “top-down” methods. Usually in “top-down” methods, the bulk material is physically or chemically broken down into smaller particles of the desired diameter. However, processes such as grinding or milling often require high temperatures and pressures, which lead to intensive energy consumption and high operation costs. In addition, chemical processes often require carcinogenic or cytotoxic chemicals, which are dangerous for humans and the environment [5].

Biological synthesis usually encourages the production of metal-containing NPs in a low-energy and environment-friendly way due to the biomolecules responsible for the synthesis and stabilization of NPs. Thus, biologically synthesized NPs are more appropriate for some applications [6,7,8]. Because of energetically efficient synthesis conditions, such as room pressure and temperature, NPs produced via fungal-assisted processes may be much more affordable. For example, Noman et al. [9] estimated the specific cost of Cu/Zn NPs (a spherical shape 51 to 100 nm in diameter) to be USD 89.8 per kg, which was significantly lower than the market price of Cu/Cn NPs synthesized with laccase enzyme, which is priced at USD 569 per kg. The authors concluded that according to their analysis, the fungal synthesis of the Cu/Zn NPs is economically feasible and may have a cost advantage over other established synthesis methods. Fungi are known to produce a large number of biomolecules that can be used in the transformation of metal ions into metal-containing NPs, provide a wide variety of centers for nucleation of the NPs, and attach to the surfaces of NPs, thereby providing stability in suspensions and unique surface properties [10,11].

When it comes to the synthesis of NPs by either ascomycetes or basidiomycetes, several factors need to be considered. These include the ease of production, potential cost of synthesis, source of the fungus or fungal metabolites, and the acceptance of consumers toward the use of mycosynthesis by certain species of fungi. Ascomycetes are well understood and industrially utilized when it comes to the production of organic compounds, e.g., *Aspergillus niger* producing citric acid [12] and *Penicillium* sp. producing antibiotics [13]. On the other hand, many species of Ascomycetes are pathogenic toward plants and animals [14,15], and the precautions that need to be applied to use NPs synthesized with these species of fungi may negatively affect the cost of the NPs. Additionally, in fields like medicine, veterinary practice, and agriculture, NPs synthesized with help of pathogenic fungi, or just fungi belonging to the same genus, may trigger both rational and irrational responses in potential buyers, which can hinder the acceptance of mycosynthesized NPs. For these reasons, basidiomycetes may play an important role [16]. Since most of the basidiomycetes used for the synthesis of NPs are either edible or medicinal, their utilization may be much more acceptable. In some instances, NPs synthesized with these fungi may have certain positive properties that result from biologically active fungal biomolecules that are adsorbed on the surface of the NPs and are not present in differently synthesized NPs. Additionally, the spent mycelia of Basidiomycetes may be a cheap and abundant source of biomolecules important for mycosynthesis since there is a large production of edible fungi grown on substrates such as sawdust [17].

Considering all these facts, the present review aimed to summarize the knowledge about the different genera (ascomycetes and basidiomycetes) that can produce NPs and to discuss the application of the synthesized NPs in various fields. The research of the last two decades was mostly concerned with the demonstration of the possibility of fungi in the production of NPs. However, there were a limited number of studies concerned with optimizing the conditions for the mycosynthesis of NPs and comparing different strains or species for NP synthesis. Additionally, the metabolites, such as the antibiotics that the fungi produce, could be effective stabilizing molecules for NPs that also have synergistic effects against certain microbes. Only a few studies compared chemically and fungal-produced NPs; in these few comparisons, the mycosynthesized NPs had either similar or slightly better performance [18]. The scientific literature also largely lacks information about upscaling the production. This lack of knowledge hinders the adoption of fungal synthesis of NPs by commercial applications.

## 2. Synthesis of Metal-Containing Nanoparticles by Fungi

**Figure 1:**
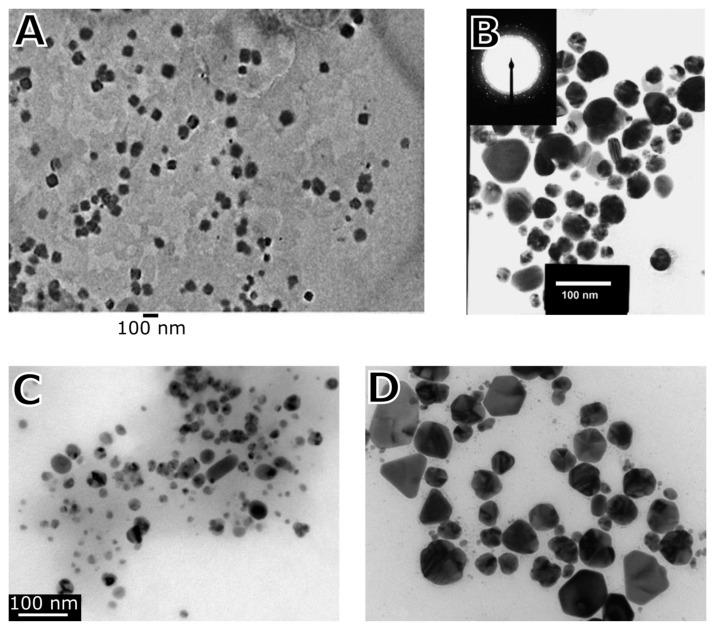
Shapes of metal-containing NPs created by mycosynthesis. (**A**) Cubic shaped Fe_3_O_4_ NPs created with filamentous fungus, *Aspergillus japonicus.* Reprinted from [34]. Copyright by Springer Nature. Reprinted with permission. (**B**) Ag NPs with the shape of hexagonal pyramids created with white rot fungus, *Phaenerochaete chrysosporium.* Reprinted from [37]. Copyright by Elsevier. Reprinted with permission. (**C**) Ag NP with spherical and rod shape created with filamentous fungus, *Trichoderma viridae*. Reprinted from [39]. Copyright by Elsevier. Reprinted with permission. (**D**) Ag NPs with circular, triangular, and hexagonal shapes synthesized with entomopathogenic fungus, *Beauveria bassiana*. Reprinted from [40]. Copyright by Springer Nature. Reprinted with permission.

The mycosynthesis of metal-containing NPs using ascomycetes and basidiomycetes is usually achieved via “bottom-up” methods, where the NPs are synthesized from the solutions containing dissolved metal ions. Instead of conventional chemicals used in NP synthesis, intracellular and extracellular pathways or the fungi cell-free extract containing the biochemically active agents such as enzymes, proteins, (poly)saccharides, etc. are used. The presence and action of these molecules can be crucial for tuning and modulating NP growth. Consequently, the reaction in this environment can dictate the shape, size, architecture, tailored functionality, and dispersity of the newly formed NPs. Apart from the use of particular species of fungi, environmental conditions such as pH of the solution, exposure time, temperature, and control of the reaction kinetics and efficiency of the NP synthesis are the most important, monitored, and optimized parameters [19]. Notably, the formation of NPs through green synthesis has some limitations. Depending on the reaction protocol and fungal species [20,21], the reaction time may or may not be slower compared with the application of a strong reducing reagent and high energy conditions [22]. Fungal extracts for the synthesis of NPs consist of a cocktail of biochemical compounds, and many of these biochemicals are active in the synthesis and stabilization of metal-containing NPs. This may lead to the formation of NPs with multiple morphologies since every active molecule in the formation of NPs may induce a different type of nucleation, and the encapsulation processes may also differ [23,24,25,26,27,28,29,30,31]. Furthermore, natural (macro)molecules capping the newly formed NPs may subsequently reduce or even prevent their further growth [32]. Most studies report the production of spherical or quasi-spherical NPs [33]. However, fungi are able to produce NPs of various shapes, including nanorods, triangular shapes, hexagonal shapes, pyramids, etc. (Table 1, Figure 1).

**Table 1:** Selected examples of fungi producing NPs of different shapes.

Species of Fungus	NP Type	Average Size (nm) and Shape	Synthesis Used	Source
*Aspergillus japonicus*	Fe_3_O_4_	82 cubic	cell-free filtrate extracellular synthesis in presence of a living fungus	[34]
*Aspergillus niger*	Fe	18 spherical	cell-free filtrate and supercritical condition of liquids	[35]
	Au	13 spherical, elliptical	cell-free filtrate	[36]
*Phaenerochaete chrysosporium*	Ag	50 to 200 hexagonal pyramids	Synthesis with mycelial mat	[37]
*Saccharomyces cerevisiae*	Pd	32 hexagonal	aqueous extract	[38]
*Trichoderma viridae*	Ag	5 to 40 spherical, rod	cell-free filtrate	[39]
*Verticillium* sp.	Au	20 spherical, triangular, hexagonal, quasi-hexagonal	intracellular synthesis in living fungus	[23]

### 2.1. Synthesis of Nanoparticles by Ascomycetes

Ascomycetes or “sac fungi” are Fungi’s phylum characteristic of the ascus, a saclike structure consisting of four to eight ascospores. The phylum includes diverse fungi, e.g., several genera of yeasts, genera of molds, *Aspergillus, Penicillium*, etc. These fungi are commonly used in the biosynthesis of organic acids, antibiotics, other organic compounds, and in the fermentation of various drink and food items [12,13,41]. Many ascomycetes, such as the genus *Trichoderma*, live in the soil and have beneficial effects on plants. Other species include edible saprophytic mushrooms, such as dead man’s fingers, truffle, and morel. Moreover, most lichens have ascomycetes as symbiotic fungi. Different genera of ascomycetes are parasitic, and this parasitism is widespread and includes parasitism on other fungi, plants, invertebrates, and vertebrates [14,15,42,43].

Generally, ascomycetes are a diverse group of fungi, and they are associated with several advantages, including their diverse survival strategies, easy manipulation with their biomass, their multiplication using simple media, their ability to tolerate high concentrations of metals, and higher productivity in terms of NP production. Thus, these organisms have been preferentially used over bacteria in many scientific studies, and the possibility to produce highly biocompatible NPs was explored [44]. Most of the studies for NP formation were based on the extracellular synthesis of NPs since it is easier to extract the NPs, and the medium is devoid of some molecules that can inhibit the growth of NPs, and the final products may not need additional treatment with detergents and ultrasound.

Most studies used just a few easily cultivable genera of fungi, i.e., *Aspergillus*, *Fusarium*, *Penicillium*, *Trichoderma*, and *Verticillium* [45,46,47,48,49]. Ascomycetes have produced many types of metal-containing NPs, including pure metal NPs of Ag, Au, and Fe [35,50,51] as well as metal oxide and sulfide NPs of Ag_2_S, Co_3_O_4_, CuO, Fe_x_O_y_, MnS, NiO, NiS, PbS, ZnO, and ZnS [35,52,53,54] (Table 2).

#### 2.1.1. Aspergillus

The members of the genus *Aspergillus* are already industrially used, and there is a large collection of knowledge regarding the growth of *Aspergillus* mycelia and how different growth parameters affect the production of the various active chemical substances this genus of fungus can produce [60]. Thus, the genus *Aspergillus* was an obvious candidate for many studies on the fungal synthesis of NPs. Bhainsa and D’Souza [61] used *A. fumigatus* to synthesize somewhat monodisperse Ag NPs that had sizes in the range of 5 to 25 nm. The fungal synthesis was found to be faster when compared with the physical and chemical approaches of NP synthesis. Similarly, *A. flavus* was also used to produce Ag NPs that were fairly monodisperse [62]. Their size ranged from 4 to 14 nm and had an average size of 9 nm. In another study, extracellular biosynthesis of Ag NPs by *A. niger* was demonstrated [33]. These mycosynthesized NPs were reported to have an average size of 20 nm, and the Ag NPs were found to have a corona made of fungal proteins. It was suggested that the Ag NPs were formed via reduction by nitrate-dependent reductase enzyme and shuttle quinone extracellular process. Endophytic *A. terreus* was used to synthesize several NPs, i.e., Co_3_O_4_, CuO, Fe_3_O_4_, and NiO NPs [52]. *A. terreus* was also observed to form Ag NPs [63].

Another class of NPs synthesized using *Aspergillus* sp. was iron oxide NPs. These were synthesized using a species of *Aspergillus*, *A. japonicus*, where 60 to 70 nm cubic-shaped magnetite NPs were produced [34]. This species of fungus was reported to hydrolyze a mixture of iron cyanide complexes, resulting in the release of ferric and ferrous ions. These compounds underwent protein-mediated co-precipitation and formed nuclei of iron oxide NPs. Another species of *Aspergillus* that was successfully used to produce NPs was *A. clavatus*. A study by Verma et al. [64] discovered that *A. clavatus* produced Ag NPs with sizes between 10 and 25 nm. Another study showed that Au NPs could be similarly produced from the extracellular extract of *A. clavatus* [65]. Extracellular synthesis of Ag NPs was also attempted with the species *A. tamarii* [66]. Their size range was 25 to 50 nm, and their shape was spherical. The saprophytic fungus *A. ustus* was isolated from the inner environment of the laboratory [67], and it was used to produce Ag NPs via extracellular synthesis. Kōji mold *A. oryzae* served as both a reducing and capping agent in the synthesis of Ag NPs. Both biomass and extracellular metabolites were able to synthesize the Ag NPs. The fungal biomass formed Ag NPs on the cell wall and also inside the cytoplasmic membrane [68]. Xerophilic *A. conicus*, isolated from plant leaves of *Avicennia marina*, *Suaeda monoica,* and *Rhizophora mucronate*, was also found to be able to produce Ag NPs. The NPs had spherical to hexagonal shapes, were polydisperse with sizes from 6 to 16 nm, and were created extracellularly [69].

#### 2.1.2. Fusarium

Several strains (i.e., saprophytes and plant pathogens) of *Fusarium oxysporum* were used to synthesize Ag NPs by Rajput et al. [70]. It was reported that all the used strains produced NPs, albeit in different quantities. It was recorded that the fungal organic molecules on the surface of NPs enhanced their stabilization in the solution. The amount of NPs produced varied depending on the strain used, and the saprophytic isolate from Denmark demonstrated the highest production of NPs. Moreover, the study noted that saprophytic fungi may be more effective at reducing aqueous metals than pathogenic strains. Durán et al. [71] indicated that extracellular production of Ag NPs by *F. oxysporum* was facilitated by reducing agents together with capping by proteins. Thus, they synthesized Ag NPs with sizes from 20 to 50 nm. Bansal et al. [72] synthesized TiO_2_ NPs using *F. oxysporum* with sizes from 6 to 13 nm. Additionally, the species was used to synthesize tetragonal BaTiO_3_ NPs with an average size of 4 to 5 nm at optimal conditions [73]. The synthesis of CdSe NPs that had an average size of 11 nm was reported by Kumar et al. [74]. In addition, Au and Cu NPs were synthesized by *F. oxysporum* [75,76]. Furthermore, a strain of *F. solani* was also used to produce Ag NPs [77]. The extracellular synthesis of the NPs led to the formation of Ag NPs ranging from 5 to 35 nm, with an average size of 16 nm. According to the FTIR analysis, there was evidence that the synthesized NPs had a capping made of fungal proteins. Other studies also reported the synthesis of Ag NPs with *F. solani* [78,79]. Furthermore, a strain of *F. semitectum* was also used to produce Ag NPs [80]. Their size varied from 10 to 60 nm, and they were spherical. The authors indicated that amino acids and peptides probably assisted the capping of the NPs and improved their stability in an aqueous medium. Ingle et al. [81] utilized another species of *Fusarium*, *F. acuminatum* to synthesize Ag NPs and evaluated their efficacy against human pathogenic bacteria. The synthesized NPs were between 5 and 40 nm in size, with an average size of 13 nm. Shelar et al. [82] selected *F. semitectum* and successfully accomplished extracellular synthesis of spherical, 8 to 50 nm-sized Ag NPs. Bawaskar et al. [83] produced Ag NPs using the plant pathogenic fungus *F. culmorum*, which ranged in size from 5 to 25 nm and had an average size of 11 nm. They further tested the inhibitory effect of these NPs in combination with antibiotics on two bacteria: *Klebsiella pneumoniae* and *Enterobacter aerogenes*.

#### 2.1.3. Penicillium

The genus *Penicillium* was considered by many studies as a suitable candidate for the synthesis of Ag NPs since the members of this genus produce a variety of extracellular metabolites capable of NP synthesis [60]. *P. citreonigrum* was used by Hamad [84] to produce Ag NPs through an extracellular approach. The resulting NPs were found to be monodisperse, spherical, and 6 to 26 nm in size. It was also observed that amide linkages and carbonyl groups were attached to the surface of these NPs, confirming the stability of NPs due to the protein capping in the aqueous medium. *P. fellutanum* isolated from coastal mangrove sediments was also utilized in the rapid synthesis of Ag NPs, and a 70 kDa protein was found to be a capping agent of the NPs [85]. Shaligram demonstrated the extracellular synthesis of Ag NPs with *P. brevicompactum*. They synthesized NPs with an average size of 58 nm and a surface coating made of proteins with primary and secondary amine groups and aromatic and aliphatic varieties. A strain of *P. verrucosum* was observed to synthesize spherical Ag NPs [86] with sizes between 3 and 24 nm. Extracellular metabolite extract of *P. notatum* was used to synthesize Ag NPs [87]. Other species of *Penicillium* that were successfully used in the synthesis of Ag NPs include *P. funiculosum*, *P. purpurogenum*, *P. nalgiovense*, *P. citrinum*, *P. italicum*, and *P. chrysogenum* [55,88,89,90,91,92,93].

In addition to Ag NPs, other NPs were synthesized utilizing the genus *Penicillium*. The species *P. aurantiogriseum*, *P. citrinum*, and *P. waksmanii* were used to produce CuO NPs of fairly spherical shape [94]. Strains of *P. brevicompactum* and *P. aculeatum* demonstrated the ability to produce Au NPs [95,96]. Melanin extract from *P. chrysogenum* was used to synthesize MgO NPs [56] and CuO NPs [57]. Additionally, *P. chrysogenum* demonstrated the ability to form ZnO NPs [18]. Extracellular metabolites of *P. aculeatum*, *P. notatum*, and *P. purpurogenome* were used to produce Zr NPs [97].

#### 2.1.4. Trichoderma

The genus *Trichoderma* is a widespread group of actinomycetes that live in the soil environment. Many of the species in the genus are opportunistic plant symbionts. Because they evolved in the soil environment and are present in most soils in the world, their metabolic apparatus was also used in the synthesis of NPs [98]. Mishra et al. [59] utilized the common soil fungus *T. viride* to synthesize Au NPs. The NPs formed within 10 min at 30 °C under optimal conditions. In addition, *T. viride* was used in the synthesis of very small (2 to 4 nm) Ag NPs [58]. Mukherjee et al. [99] utilized *T. asperellum* to synthesize Ag NPs with a size range of 13 to 18 nm via extracellular synthesis. Devi et al. [100] used 75 strains of five species, i.e., *T. asperellum*, *T. harzianum*, *T. longibrachiatum*, *T. pseudokoningii*, and *T. virens* to produce Ag NPs. NPs with sizes of 8 to 60 nm were produced. Nitrate reductase activity was highest in *T. virens* VN-11, and this strain was also able to produce the highest number of Ag NPs. Ahluwalia et al. [101] used *T. harzianum* to produce Ag NPs ranging in size from 20 to 100 nm, with 51 nm NPs being the most frequently measured. *T. reesei* was also used to synthesize Ag NPs. Vahabi et al. [102] synthesized Ag NPs with a size range of 5 to 50 nm utilizing *T. reesei*. Gemishev et al. [103] produced Ag NPs that had sizes from 15 to 25 nm. The NPs had a capping made of fungal biomolecules. *T. harzianum* was also utilized in the formation of ZnO, Cu, and hematite (Fe_2_O_3_) NPs [104,105,106]. A strain of *Trichoderma atroviride* was used to produce Cu NPs with a size range of 5 to 25 nm [107].

#### 2.1.5. Verticillium

The genus of filamentous fungi *Verticillium* was one of the first used in the synthesis of NPs. Mukherjee et al. [23,108] demonstrated that *Verticillium* could produce Ag and Au NPs from their respective dissolved chloride salts. Later, Gericke and Pinches [109] synthesized Au NPs under varying pH conditions with the help of *V. luteoalbum*. The changes in pH affected the shape, size, and number of NPs. Soni and Prakash [48] also synthesized spherical Au NPs and Ag NPs using *V. lecanii* and evaluated their effectiveness in mosquito control.

#### 2.1.6. Yeasts

Like filamentous fungi, yeasts have been reported to have the ability to accumulate relatively high concentrations of metal ions from the surrounding environment. Therefore, they have also been explored for the synthesis of metal-containing and other inorganic NPs [5]. The psychrotrophic yeast, *Yarrowia lipolytica*, was used to produce Ag NPs [110]. The authors suggested that melanin molecules were crucial for the biogenic mineral formation of the Ag ions into NPs. Similarly, Waghmare et al. [111] produced Ag NPs extracellularly utilizing *Candida utilis*. The synthesized NPs were found to be spherical in shape and had sizes between 20 and 80 nm. Eugenio et al. [112] used *Candida lusitaniae* to produce Ag NPs. They produced smaller NPs with a size range of 2 to 10 nm. Oropharyngeal *Candida glabrata* was also utilized in the production of spherical Ag NPs with sizes between 2 and 15 nm [113]. Genetically modified yeast, *Pichia pastoris*, with overexpression of the cytochrome b5 reductase enzyme gained from *Mucor racemosus*, was also found to reduce Ag ions to Ag NPs. This yeast produced larger Ag NPs that ranged from 70 to 180 nm [114]. Other species of yeasts that were employed in Ag NP synthesis include *Paecilomyces lilacinus*, *Lecanicillium lecanii*, and the ubiquitously used *Saccharomyces cerevisiae* [115,116,117]. NPs other than Ag NPs were also produced by yeasts. Sriramulu and Sumathi [42] used *S. cerevisiae* to produce extracellular metabolites for the synthesis of hexagonal Pd NPs of an average size of 32 nm. *S. cerevisiae* is also capable of producing MnO_2_ NPs, as was documented by Salunke et al. [118]. Zhang et al. [119] reported the synthesis of Au NPs by the yeast *Magnusiomyces ingens*. Kowshik et al. [120] synthesized small (1 to 1.5 nm) CdS NP inside cells of *Schizosaccharomyces pombe*. Similarly, Dameron et al. [121] utilized *C. glabrata* and *S. pombe* to intracellularly synthesize 2 nm CdS crystallites and 2.9 nm extracellular CdS crystallites. They reported that intracellular CdS crystallites were capped by γ-glutamyl peptides, and the CdS intracellular crystallites were more monodisperse than the CdS crystallites produced by some chemical methods at that time.

#### 2.1.7. Other Ascomycetes

Many species of ascomycetes other than above mentioned five genera have been used in the synthesis of NPs. The ability of pathogenic *Scopulariopsis brumptii* to produce Ag NPs by extracellular synthesis was investigated [84]. The produced NPs had sizes between 4.2 and 23 nm, and they were applied to polyurethane foam to discern their antimicrobial activity. The surface of the NPs was covered with fungus-derived biomolecules with amide linkages and carbonyl groups that suggest that they were protein-stabilized. Birla et al. [122] employed extracellular metabolites of the pathogenic fungus *Phoma glomerata* in the synthesis of Ag NPs. Rai et al. [123] used three species of *Phoma*, i.e., *P. capsulatum*, *P. putaminum*, and *P. citri*, to form Ag NPs. All three species produced NPs with similar size ranges of 5 to 90 nm. Even an entomopathogenic fungus, *Beauveria bassiana*, was used to synthesize Ag NPs with a size range of 37 to 61 nm [124]. Irregularly shaped Ag NPs with an average size of around 40 nm were synthesized using an extract from *Acremonium diospyri* [125]. *Pestatolia* sp. isolate from the leaves of *Syzygium cumini* was used for the synthesis of Ag NPs [126]. Spherical polydisperse NPs of 10 to 40 nm (with an average size of 12 nm) were created. *Cladosporium cladosporioides* was used to synthesize protein-coated Ag NPs with a size range of 10 to 100 nm [127]. Extract synthesis, or extracellular or intracellular synthesis by several other species, i.e., *Alternaria solani*, *Colletotrichum incarnatum*, *Curvularia nodulosa*, *Epicoccum nigrum*, *Neurospora crassa*, *Neurospora intermedia*, and *Nigrospora oryzae,* was used in the formation of biologically formed Ag NPs [88,128,129,130,131,132].

The soil fungus *Cladosporium oxysporum* was selected by Bhargava et al. [133] to synthesize Au NPs extracellularly. Mishra et al. [70] utilized culture filtrate of *Hypocrea lixii* to produce Au NPs. *N. crassa* was also observed to intracellularly synthesize Au NPs as well as bimetallic Ag/Au NPs [132]. The same species of fungus also produced Pt NPs of various sizes depending on the protocol used [134]. In another study by Li and Gadd [135], Cu carbonate in the form of malachite with a trace amount of azurite was produced by *N. crassa*. Additionally, Al_2_O_3_ NPs were produced by fungal synthesis. Suryavanshi et al. [136] utilized *Colletotrichum* sp. in their production, and the NPs were stabilized by essential oils extracted from the *Eucalyptus globulus* and *Citrus medica*.

### 2.2. Synthesis of the Nanoparticles with Basidiomycetes

Most common edible and medicinal mushrooms form a fruiting body and belong to the phylum Basidiomycota. The mushroom’s fruiting body is consumed and valued for its sensory, health-promoting, beneficial properties in the human diet, and it contains basidia characteristics for sexual spore reproduction [137]. Besides biomedical and nutritional properties, recent studies focused on the possibility of basidiomycetes’ application in green chemistry, as they can synthesize various types of metal-containing NPs [16]. The anticancer effects of Ag and Au NPs synthesized using *Pleurotus sajor-caju* were investigated as a new potential drug [138]. The authors also demonstrated the presence of pleuran, the active beta-glucan molecule essential in *Pleorotus* sp., on the surface of synthesized Ag NPs. Rabeea et al. [139] for the first time used the fruiting bodies of *Flammulina velutipes* to synthesize the Au NPs, which were successfully used as a catalyst in the decolorization and degradation of methylene blue dye. In soil mycoremediation, metal contaminants can be potentially stabilized through similar processes of metal reduction as observed for metal-containing NP synthesis. A spent mushroom substrate, a product of edible mushroom production, can be reused with/without NPs as a new soil bionanoremediation tool [140].

**Figure 2:**
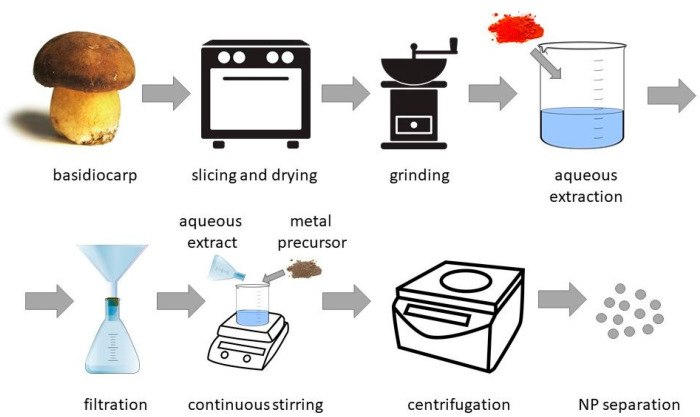
Graphical illustration of mycosynthesis of metal-containing NPs by basidiomycetes.

Different parts of basidiomycetes, such as mycelia or fruiting bodies, can be used for NP synthesis. The content of active chemicals in biosynthesis can differ between the mushroom’s fruiting body and the mycelia. The level of metals such as Cd, Pb, or Hg is higher in the fruiting body. The efficient concentration or so-called “biofortification” of essential ions such as Zn, Li, and Fe is also more effective in fruiting bodies [137]. Moreover, the mycelia of *Agaricus* sp. mushrooms typically had lower concentrations of antioxidants such as phenolic acids [141]. Mushroom antioxidants are possibly involved in the NP-forming reactions. One of the typical processes of NP synthesis mediated with aqueous extracts of basidiomycetes can be viewed in Figure 2.

Mycosynthesis of metal-containing NPs by macrofungal species such as *Pleurotus* sp., *Agaricus* sp., *Ganoderma* sp., or *Letilus* sp. has recently gained enormous attention since these mushrooms are common and safe to use for humans and the environment. They are well-known and well-described. Moreover, they are non-toxic, non-pathogenic, and easy to cultivate via submersion and grow at a large scale [16]. Green synthesis using basidiomycetes extract containing NP-forming compounds was demonstrated for extracellular and intracellular synthesis of NPs.

In the study of Vetchinkina et al. [142], NPs of Au and Ag, as well as NPs containing the non-metals Se and Si were successfully synthesized via extracellular and intracellular approaches using *P. ostreatus*, *L. edodes*, *G. lucidum,* and *G. frondosa*. They demonstrated that the synthesis of these NPs was directly linked to mushroom metabolism, which strongly depends on the cultivation conditions. The study concluded that the reduction of HAuCl_4_ and AgNO_3_ to Au and Ag NPs was due to activity of phenol oxidases, Mn-peroxidases, laccases, and tyrosinases. On the other hand, the synthesis of non-metals—Si- and Se-containing NPs—did not depend on phenol oxidase activity but was dependent on different types of enzymes, i.e., NADH-dependent nitrate and nitrate reductases. Selected examples of metal-containing NPs synthesized by various species of basidiomycetes can be viewed in Table 3.

#### 2.2.1. Pleurotus

The oyster type of mushroom (*Pleurotus* sp.) is the most common species of basidiomycetes used in the mycosynthesis of nanomaterials. The most popular oyster-synthesized metal-containing NPs include Ag NP and Au NP, which are popular due to their biomedical (anti-cancer), antimicrobial, and other applications [16]. Ag NPs were mycosynthesized for the first time using protein isolated from *Pleurotus ostreatus* spent mushroom substrate in 2007 [62]. Moreover, various studies described and proved the excellent antimicrobial properties of Ag NPs synthesized by *Pleurotus sajor-caju* against Gram-positive and Gram-negative bacteria [149,150]. In another study, Ag NPs of an average size of 7 nm were formed as the result of a one-hour-long photo-biochemical reaction combining the mushroom extract of *Pleurotus citrinopileatus* with an AgNO_3_ solution. Results of the study suggested that photosensitive flavoproteins enhanced the reduction of Ag(I) ions into Ag(0) since the reaction in dark conditions was 1.8 times longer compared with the reaction exposed to sunlight irradiation [32].

The review by Bhardwaj et al. [151] summarized different methods of metal NP synthesis from metal ion solutions, their applications, and the recent progress using various types of the *Pleurotus* sp. In the synthesis process, reducing and stabilizing agents present in different parts of the mushroom, such as the fruiting body or mycelia, can be used. The active aqueous extract is usually prepared by soaking and boiling the basidiocarps in distilled water, followed by filtration of the biomass residue. Cell-free extracts used for the NP synthesis have an added advantage because the formed NPs do not need to be separated from the fungal biomass, making the process easier and more effective [142].

In the presence of sunlight, the *P. florida* fruiting body filtrate was used for Au NP production by the photo-exposition method [152]. ZnO NPs were synthesized from the Zn(NO_3_)_2_ water solution using *P. djamor* extract in a 1:4 volume ratio [153]. The filtrate of *P. sapidus* was used to synthesize Au NPs, which ranged from 15 to 100 nm in size [154].

#### 2.2.2. Agaricus

The second most commonly used edible mushroom genus for NP synthesis is *Agaricus* sp., and the species *A. biporus*, commonly known as button mushroom, is particularly common [155]. *Agaricus* is a saprophytic soil basidiomycetes that degrades various types of metal molecules by secreting hydrolytic enzymes. In addition to enzymes, the fungal extract contains terpenoids, tannins, polysaccharides, phenolic compounds, and flavonoids, which act as reducing and stabilizing agents in the synthesis of metal-containing NPs [156]. Moreover, not just the fruiting body but even mycelia can be used as a reducing and stabilizing agent, as published in the study by Loshchinina et al. [157]. The authors used cell-free filtered intracellular and extracellular extracts of *A*. *bisporus* and *A. arvensis* to produce Au NPs and Ag NPs using substrates with different starting concentrations of metal salts [157]. Au NPs were also synthesized using *A. bisporus* extract through a hydrothermal process, and the FTIR analysis showed that polyols and carbonyl groups had a strong effect on the formation of stable Au NPs [156].

#### 2.2.3. Ganoderma

Lingzi or reishi, the medicinal mushroom of *Ganoderma* sp., has over 250 varieties identified, and some of them have already been used to produce metal-containing NPs [158]. The extract of *G. lucidum* fruiting body contained phenolic, flavonoid, and polysaccharide compounds, which were considered to be the active agents in Ag NP synthesis and were also considered important for their resultant antioxidant and bactericidal activity [159]. Dried powder of *G. applanatum* and *G. lucidum* fruiting bodies was used to prepare an aqueous extract and was mixed with a silver nitrate solution to prepare Ag NPs of various sizes, which were then tested as nanomedicine in cancer treatment [147,160].

Ag NPs that are stable in water were prepared using hot aqueous mycelia extract of *G. neo-japonicum,* and the NPs were tested against breast cancer cells [161]. In a different study, the Ag NPs obtained with the extract of *G. sessile* had a smaller size compared with Ag NPs obtained by ascomycetes *Trichoderma harzianum*. Both mycosynthesized Ag NPs had no detected cytotoxic effect on human cell viability, inhibited bacterial growth, and had a storage life of over one year [162]. Spherical Au NPs were synthesized using the extract of *G. lucidum* and were microwaved to speed up the synthesis. The average size of the NPs was 22 nm, which was smaller than those generated with the conventional heating method [163].

## 3. Application of Fungal-Synthesized Nanoparticles

**Figure 3:**
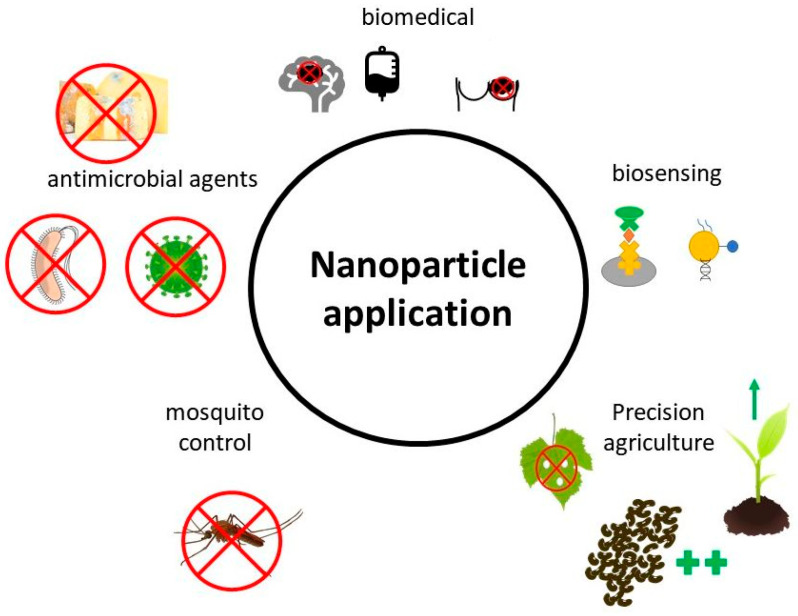
Different applications of metal-containing NPs created by mycosynthesis.

Fungi seem to be very appropriate candidates for NP synthesis. They produce a large number of enzymes and metabolites that are capable of NP formation, and there is a precedence for their use in other fields, such as the production of organic acids by *Aspergillus* sp., pharmaceutical fine compounds by *Penicillium* sp., industrial production of enzymes such cellulase, hemicellulase, and cellobiohydrolase by *Trichoderma* sp., and the many species used in food fermentation and food production. The biomass waste from these industries could be used for the cost-effective biosynthesis of NPs [18,60]. This section of the review is concerned with fungi-synthesized NPs that have been experimentally applied for various uses including biomedical applications, catalysis, biosensing, antimicrobial agents, insecticides, precision agriculture as nanofertilizers and nanopesticides, environmental, and remediation applications (Figure 3).

### 3.1. Biomedical Applications

Fungal synthesis may help produce NPs that are more benign to human tissues since the process does not involve toxic compounds, and the biomolecules that stabilize the NPs may increase biocompatibility. *F. solani* isolated from the plant *Chonemorpha fragrans* was used in the synthesis of Au NPs with the size of 40 to 45 nm. Their anti-cancer efficacy was tested on cervical cancer cells (He La) and against human breast cancer cells (MCF-7), and the obtained results showed dose-dependent toxicity. The NPs induced cell apoptosis. The authors suggest that mycogenic NPs are a safer alternative to conventional chemotherapeutic agents [164]. *Penicillium brevicompactum* extracts were used in Au NP synthesis. The NP size was positively correlated with the initial concentrations of Au ions. The optimized method was used to synthesize NPs with a size range of 20 to 50 nm. These NPs were then applied to mouse cancer C_2_C_12_ cell lines. The resulting cytotoxicity showed a time-related and dose-related effect [96].

Arun et al. [165] synthesized Ag NPs that were applied to Human Epidermoid Larynx Carcinoma (HEP-2) cell lines. In the experiment, 27.2 and 64% cell mortality were recorded at concentrations of 10 and 100 µg∙mL^−1^, respectively. Ag NPs synthesized by the endophytic fungus *Colletotrichum incarnatum* inhibited thrombin activity and generation. Additionally, the NPs had an antibiotic effect against biofilm-forming Gram-positive (*Vibrio cholerae*) and Gram-negative bacterium (*Bacillus cereus*). The Ag NPs had high cytotoxicity against human cancerous (HeLa) cells at 50 μg∙mL^−1^, while the cytotoxicity against human epithelial cells was low. The authors suggest that Ag NPs can be used for managing acute thrombosis and also in other biomedical applications [128]. Well-dispersed Ag NPs extracellularly synthesized by *Guignardia mangiferae* were used by Balakumaran et al. [166] to study cytotoxic effects on HeLa (cervical) and MCF-7 (breast) cells; the IC50 values for these cell lines were 27.54 and 23.84 μg∙mL^−1^, respectively. Husseiny et al. [167] measured the antitumor potential of Ag NPs formed using *F. oxysporum*. The NPs were highly inhibitive towards a tumor cell line of MCF-7 cells (human breast adenocarcinoma). The effect of NPs was ascribed to the disruption of the mitochondrial respiratory chain, reactive oxygen species generation, and the hindered synthesis of adenosine triphosphate (ATP), which caused damage to DNA. Syed et al. [54] discovered that α-Ag_2_S NPs produced by extracellular synthesis were effective against human breast cancer cell line (ZR-75-1) and human Burkitt’s lymphoma cancer (Daudi).

### 3.2. Antimicrobial Agents

Due to their small size, NPs are readily accessed by cells and are capable of harming bacteria, fungi, and viruses due to the toxicity of the chemical compounds that comprise them as well as through nano-specific toxicity caused by their large surface area, which may have a high energy potential and can generate a large number of reactive oxygen species.

#### 3.2.1. Antibacterial Agents

NPs obtained from sustainable sources can potentially be inexpensive and environmentally and medically safe options for the treatment of fungal infections, enabling the control of fungi resistant to conventional antibiotics [168]. They may be used in cases where there are multi-resistant strains of bacteria. Most of the antimicrobial NPs synthesized with fungi have been Ag NPs. Durán et al. [169] demonstrated the synthesis of small Ag NPs (1.6 nm) using *F. oxysporum.* Furthermore, these NPs were incorporated into clothes and used to prevent infection from *S. aureus*. Ag NPs synthesized using *F. oxysporum* were also utilized by Srivastava et al. [170] for assessing their antimicrobial efficacy, and they were found to be effective antimicrobial agents against *Escherichia coli* and *Pseudomonas aeruginosa* in-silico and in-vitro. Similarly, El-Rafie et al. [78] reported the antimicrobial effect of Ag NPs applied on cotton fabric. The NPs imparted the textile with a 97% and 91% reduction of *S. aureus* and *E. coli*, respectively. Namasivayam and Avimanyu [116] reported an improved antibiotic effect with Ag NPs synthesized with *Lecanicillium lecanii*. The NPs increased the effect of several antibiotics against *S. aureus* and *E. coli*. They proposed that the Ag NPs can be utilized in bandages and other medical fabrics to prevent severe wound infections. Datta and Desay [87] reported the synthesis of Ag NPs using *Penicillium notatum*. These NPs were applied to paper to design antimicrobial wipes for hospitals. The Ag NPs were effective against *E. coli*, *Streptococcus pneumoniae*, *S. aureus*, *B. cereus*, and *B. subtilis*. Apte et al. [110] used Ag NPs synthesized by yeast *Yarrowia lipolytica* against bacterial biofilms. They found their biosynthesized Ag NPs to be effective against the biofilms of *Salmonella paratyphi*. Ag NPs were effective against *E. coli*, *Streptococcus pneumoniae*, *S. aureus*, *B. cereus*, and *B. subtillis* in the study. There are many other studies that employed Ag NPs against human pathogenic microbes and found high antimicrobial activity [66,84,88,101,111,115,122,126,165,171,172,173].

The filamentous fungus *Colletotrichum plurivorum* was used for the synthesis of Ag_2_O nanocuboids. These NPs were employed against the pathogenic Gram-negative bacterium *E. coli* and Gram-positive bacterium *B. subtilis,* and they substantially inhibited their growth [174]. Gupta and Chundawat [75] produced Cu NPs with extracellular synthesis using *F. oxysporum* to evaluate their antimicrobial activity. They were used against *P. aeruginosa*, *E. coli*, *K. pneumoniae*, and *S. aureus.* Naimi-Shamel et al. [76] synthesized Au NPs with *F. oxysporum* and conjugated the NPs with tetracycline, and the combination increased the antimicrobial effect against Gram-positive and Gram-negative bacteria. Islam et al. [175] synthesized AuSe NPs using the endophytic fungus *F. oxysporum.* The NPs were applied against *A. niger*, a common ubiquitous fungus, and they displayed antisporulant activity. This antisporulant property of Au Se NPs could have implications for the control of the growth of mycelia and could thus increase industrial productivity. Additionally, NPs can be used to minimize the chance of fungal re-infections. Sharma et al. [176] created ZnO NPs using the white-rot fungus *Phanerochaete chrysosporium*. The ZnO NPs were then impregnated with a single step in cellulose fibers. The antimicrobial activity of these fibers was then observed and evaluated. The ZnO NPs significantly inhibited the growth of two bacteria species: *E. coli* and *S. aureus*.

#### 3.2.2. Antifungal Agents

Fungal-produced Ag NPs have also been employed as antifungal agents. The previously mentioned ZnO NPs produced by Sharma et al. [176] also displayed antifungal activity against *A. niger*, *Geotrichum candidum*, and *P. chrysosporium*[172]. Win et al. [177] used the fungus *Alternaria* sp. isolated from banana-cultivated soil to produce 3 to 10 nm-sized Ag NPs. They were shown to have the ability to reduce the growth of the fungi *Alternaria* sp. and *Fusarium* sp. Pereira et al. [55] employed Ag NPs synthesized with *P. chrysogenum* and *A. oryzae* against strains of dermatophytic pathogenic fungus *Trichophyton rubrum*. The Ag NPs had high antifungal activity that was comparable to conventional antifungal agents. Culture supernatants of *Aspergillus sydowii* were also used to synthesize Ag NPs [178]. The Ag NPs were then applied against 12 strains of fungi from the genera *Aspergillus*, *Candida*, *Cryptococcus*, *Fusarium*, and *Sporothrix*. The NPs showed high antifungal activity toward all tested fungus strains at a relatively low concentration. A marine fungal consortium filtrate of *Penicillium oxalicum* and *Fusarium hainanense* was used to synthesize 5 nm Ag NPs [179]. These Ag NPs had better antifungal activity against *F. oxysporum* than ampicillin. The cell-free supernatant of *P. chrysogenum* was used to synthesize CuO NPs with an average size of 10 nm via gamma-ray irradiation [57]. The CuO NPs had high antifungal activity against *A. solani*, *A. niger*, and *F. oxysporum*. Similarly, 10 nm-sized MgO NPs created by *P. chrysogenum* melanin pigment were employed against *Candida albicans* and were found to be a promising antifungal agent [58]. Hexagonal 34 to 55 nm ZnO NPs were synthesized using a fungal extract of *Xylaria acuta* [180]. The ZnO NPs exhibited antifungal activity against the plant pathogens *F. oxysporum* and *Phomopsis* sp. as well as the common contaminants *Aspergillus flavus* and *Cladosporium cladosporioides.* This antifungal activity showed a dose-dependent effect with a high percentage of mycelial inhibition.

Another common approach in the synthesis of nanoparticulate antifungal agents is to use NPs in combination with standard organic antifungal molecules. Gajbhiye et al. [181] tested Ag NPs produced with extracellular extract of *Alternaria alternata* cell filtrate in combination with fluconazole against *C. albicans*, *Fusarium semitectum*, *Phoma glomerata*, *Phoma herbarum*, and *Trichoderma* sp. The combination of Ag NPs with fluconazole significantly increased the fold area of inhibition of *C. albicans*, *P. glomerata*, and *Trichoderma* sp.; however, no significant increase in the fold area of inhibition was observed in *F. semitectum* and *P. herbarum*.

#### 3.2.3. Antiviral Agents

Gaikwad et al. [182] mycosynthesized Ag NPs utilizing the fungi *Alternaria* sp., *F. oxysporum*, *Curvularia* sp., *Chaetomium indicum*, and *Phoma* sp. against herpes simplex virus and human parainfluenza virus type 3. While *F. oxysporum*, *Curvularia* sp., and *C. indicum* had the highest effectiveness and low cytotoxicity, the other two types were not used due to their higher human cytotoxicity.

### 3.3. Catalysis

Few studies have used mycosynthesized NPs in catalytic applications. Sriramulu and Sumathi [42] synthesized Pd NPs using yeast extract of *S. cerevisiae*. They then applied the NPs in photocatalysis of direct blue 71 dye, where 98% of the dye was degraded within 1 h and about 70% was degraded within the first 10 min. Elegbede et al. [171] synthesized Ag NPs with xylanases of *A. niger* and *T. longibrachiatum*. They observed scavenging of DPPH (37.5 to 79.4%) and hydrogen peroxide (20.5 to 96.5%) by Ag NPs, and Ag NPs were shown to be effective in the degradation of malachite green (79.0%) and methylene blue (25.3%). Pei et al. [183] used the fungus *Mariannaea* sp. isolated from activated sludge in the laboratory, and the synthesis with living cells of mycelia was found to produce Au NPs with an average size of 37 nm while the extract was reported to produce Au NP with an average size of 12 nm. Both Au NPs were excellent catalysts of 4-nitrophenol. Bhargava et al. [133] synthesized Au NPs using the soil fungus *Cladosporium oxysporum*. The NPs had a quasi-spherical shape with an average size of 72.3 ± 21.8 nm. These Au NPs displayed high efficiency in the reduction of rhodamine B mediated by sodium borohydride, NaBH_4_, within 7 min. The encapsulation by proteins was given as an explanation of the high efficiency of catalysis. Mishra et al. [59] produced Au NPs using cell-free extracts of *T. viride* and *Hypocrea lixii*. The mycosynthesized NPs were an excellent catalyst that reduced 4-nitrophenol to 4-aminophenol in the presence of NaBH_4_. Qu et al. [184] produced small Au NPs using cell-free *Trichoderma* sp. extracts. The NPs were spherical in shape and had an average size of 10 nm with a size range of 1 to 24 nm. These NPs had a good catalytic efficiency for 2-nitrophenol, 3-nitrophenol, 4-nitrophenol, 2-nitroaniline, and 3-nitroaniline. They also had high catalytic efficiency for discoloration of azo dyes.

### 3.4. Biosensing

Zinc sulfide quantum dots (ZnS QDs) were produced using cell-free extracts of *Aspergillus* sp. under ambient conditions [185]. The spherical crystalline ZnS QDs had a mean diameter of 6.3 nm. The NPs were investigated for photodegradation of the dyes methylene blue, crystal violet, and Congo red, and for sensing of heavy metals in the aqueous phase. The photodegradation of the dyes resulted in a 70 to 80% reduction in the dyes after 6 h. The mycosynthesized ZnS QDs were found to be good at sensing Pb(II), and they could potentially be tuned to sense Zn(II), Cd(II), and Cr(II). Priyanka et al. [186] synthesized PbS NPs with the endophytic fungus *A. flavus* to detect arsenic in water. The NPs had a size range of 35 to 100 nm and were able to detect As(III) at a concentration of 50 µg∙L^−1^. There was no interference from other ions at concentrations of up to 20 µg∙L^−1^.

### 3.5. Mosquito Control

Some entomopathogenic fungi, such as *F. oxysporum* or *Chrysosporium keratinophilum*, have been used to synthesize mosquito-controlling agents [187,188]. Based on these applications, these and other related fungi have also been used in the synthesis of NPs to control the spread of mosquitos. Kamalakannan et al. [86] used *Penecillium verucosum* to produce Ag NPs. These NPs were applied against the mosquito *Culex quinquefasciatus* and were found to have a significant negative effect on the development of mosquito larvae and pupae. The entomopathogenic fungus, *Beauveria bassiana*, was used by Banu and Balasubramanian [124] to synthesize Ag NPs. These NPs were then applied against the different larval instars of *Aedes aegypti* at different concentrations for a period of 24 h. The NPs significantly reduced the development of several stages of the mosquito and proved to be a good alternative for their potential control.

### 3.6. Precision Agriculture—Nanofertilizer and Nanopesticide Applications

Although many different NPs have been used in agricultural research to produce nanofertilizers and nano-growth-enhancers, there are only a few reports on mycosynthesized NPs applied to plants.

Raliya et al. [189] synthesized ZnO NPs using *A. fumigatus,* and these NPs were applied to soil to enhance P uptake by mung bean (*Vigna radiata*). The application of the NPs increased the mobilization of P by 10.8%. The activities of phosphatase and phytase enzymes were also increased by 84 and 108%, respectively. Mycosynthesized ZnO also positively affected plant phenology, i.e., stem height, root volume, and biochemical indicators such as leaf protein and chlorophyll contents. Fouda and Sofy [18] found that ZnO NPs can be produced using *P. chrysogenum* cell-free extracts. The mycosynthesized NPs were compared to chemically synthesized NPs and their ionic equivalent, ZnSO_4_. Out of the three applications, the mycosynthesized NPs showed a similar or more beneficial effect on growth and production parameters, such as photosynthetic characteristics, levels of soluble sugars, proteins, phenols, oil content, and mineral concentration as compared with the ionic Zn and chemically synthesized ZnO NPs. Shobha et al. [190] synthesized ZnO NPs with soil-extracted strains of *Trichoderma* sp. The antimicrobial activity against the pathogen causing bacterial leaf blight in rice, *Xanthomonas oryzae*, was tested, and the NPs were found to reduce the growth of the bacteria. The highest inhibition was reported in a case of co-cultured strains of *Trichoderma*. *T. harzianum*-mediated ZnO NPs were utilized in a study by Zaki et al. [191] for inhibition of the soil-born pathogenic fungi *Fusarium* sp., *Rhizoctonia solani*, and *Macrophomina phaseolina*. The NPs completely reduced the growth of these pathogens in vitro as well as significantly inhibited cotton seedling disease symptoms under greenhouse conditions.

There have been several studies on the use of Ag NPs for the control of phytopathogenic fungi in agriculture. *A. versicolor* was used by Elgorban et al. [192] to synthesize Ag NPs to control the phytopathogenic fungi *Sclerotinia sclerotiorum* and *Botrytis cinerea* in strawberry plants. The NPs displayed concentration-dependent toxicity toward both fungi and had a higher inhibitory effect against *B. cinerea*. Mycosynthesized Ag NPs utilizing *Epicoccum nigrum* were used against the fungal pathogens *A. flavus*, *A. fumigatus*, *Candida albicans*, *Cryptococcus neoformans*, *F. solani*, and *Sporothrix schenckii* [130]. In another study, the fungus *Guignardia mangiferae* was used to produce Ag NPs that were able to inhibit the growth of phytopathogens such as *Colletotrichum* sp., *Curvularia lunata*, and *Rhizoctonia solani* [166]. El-Aziz et al. [193] used the phytopathogenic fungus *F. solani* that was isolated from wheat to produce Ag NPs that were able to control the spread of other grain-born phytopathogenic fungi.

Other studies combined biosynthesized Ag NPs with conventional biocides. An effect of fluconazole with Ag NPs synthesized with the fungus *Alternaria alternata* was evaluated by Gajbhiye et al. [181]. Potentiation of fluconazole was observed for *P. glomerata* but not for *F. semitectum* and *P. herbarum*. Ag NPs biosynthesized using *T. harzianum* were applied with triclabendazole to control the *Fasciola* sp. parasite, a common parasite of sheep and cattle. The comb ination of Ag NPs with the drugs was 20% more effective against *Fasciola* sp. eggs than the drug alone, which produced 70.6% inhibition. The combination of Ag NPs with the drug thus may have the potential to overcome the resistance parasites have acquired against the triclabendazole [194]. Guilger-Casagrande et al. [195] synthesized Ag NPs using *T. harzianum* cultivated with (AgNP-TS) and without enzymatic stimulation (AgNP-T) by the cell wall of *Sclerotinia sclerotiorum*. Their activity against the plant pathogenic fungus *Sclerotinia sclerotiorum* was then evaluated. Both types of NPs showed high inhibitory effects against the plant pathogenic fungus, with AgNP-TS causing a higher reduction in mycelial growth.

Moreover, Sawake et al. [196] recently demonstrated the synthesis of CuO NPs using cell-free extracts from *Pseudomonas fluorescens* and *T. viride,* and they evaluated their antifungal effect against *Phytophthora parasitica*, a common fungus causing gummosis in citrus. The *T. viride*-synthesized CuO NPs showed higher inhibition of the pathogenic fungus, and in vivo experiments indicated a significantly higher antifungal activity than the traditionally used Bordeaux mixture.

## 4. Conclusions—Advantages, Limitations, and Future Prospects of Fungal Synthesis of Metal-Containing Nanoparticles

A significant advancement in the area of fungus-produced NPs and their application was observed in the last two decades [5]. Consequently, the synthesis of metal-containing NPs by fungi may be a more convenient and environmentally friendly way of producing NPs. The reported NP synthesis pathways in the review were of laboratory scale. However, there are precedents for large-scale mycosynthesis of metal-containing NPs. Many production processes using fungi to generate antibiotics, organic acids, and other organic compounds exist. They can be adjusted to produce NPs, and the waste from the production of these compounds can be potentially used to produce NPs [60]. Additionally, large-scale production of biofabricated NPs has been attempted using other organisms, such as the bacterium *Thermoanaerobacter* sp. TOR-39 to synthesize Zn-substituted magnetic NPs [197], CdS NPs [198], and ZnS NPs [199]. Moreover, Te(0) NPs were synthesized using toxic tellurite oxyanions (Te(IV)) and methanogenic microbes [200]. Because these kinds of reactors were able to produce NPs on a larger scale and because we have been producing organic compounds via fungal synthesis on an industrial scale for decades, the possibility of creating efficient, scalable protocols for fungal NP production is very high.

### 4.1. Advantages and Limitations

The fungal production of metal-containing NPs also has a few advantages over other production methods. One of the often-discussed benefits is that the biological synthesis process is often free from toxic chemical contaminants essential for production via physicochemical processes. This trait is crucial for biomedical and agricultural applications. The other benefit is that the output of the mycosynthesized NPs is often a one-step process, where the fungal biomolecules play a role not only as templates or agents in the synthesis of NPs but can also act as capping agents thereby stabilizing the NPs. Moreover, selective use of certain species of fungi may lead to capping with medically or agro-chemically essential molecules that will provide additional biomedical or agricultural effects, thus increasing the efficiency of the mycosynthesized NPs [5]. Furthermore, fungal NP synthesis can be effectively adjusted with the use of different fungal biomolecules. Birla et al. [201] reported that under the right conditions, *F. oxysporum* extracts can synthesize Ag NPs within 5 min if the medium is exposed to sunlight irradiation. Abu-Tahon et al. [202] found that Au NPs can be synthesized within 2 min of the application of chloroauric acid to the extract of *A. flavus*.

Synthesis of metal-containing NPs via fungal mediation is not without challenges that need to be overcome. The efficiency of NP synthesis needs to be improved, and the control over the crystallinity, particle size, dispersity, and morphology of the mycosynthesized NPs needs to be standardized in a reproducible manner. Nevertheless, several studies have shown the production of monodisperse NPs via alternating environmental conditions such as growth medium type, pH, temperature, concentrations of precursors, variety and amount of biomass, and irradiation with sources of different wavelengths, e.g., gamma rays, UV, visible, or microwave. As stated earlier, Birla et al. [201] produced monodisperse Ag NPs rapidly under optimal conditions. Other research studies also succeeded in using fungal synthesis to produce monodisperse Ag NPs [203]. Eldomany et al. [204] were able to synthesize Au NPs with fair monodispersity using extracts from the edible mushroom *Pleurotus ostreatus*.

### 4.2. Future Prospects

There was an immense increase over the last two decades in studies on the fungal synthesis of NPs for various agricultural, biomedical, catalytic, and biosensor applications. The ability to adjust the NP properties by slightly changing some of the synthesis parameters, together with the stabilization by biomolecules from the fungi that can serve specific purposes, gives mycosynthesized NPs an advantage over conventionally synthesized NPs. Due to their large surface-area-to-volume ratio, antibacterial, antioxidant, or optical attributes combined with benign or bioactive fungal capping agents, the fungus-mediated synthesis of NPs can be an essential technology in the future. However, there are still a lot of knowledge gaps that prevent us from fully utilizing the tremendous abilities of fungi to produce the biomolecules that can effectively synthesize and stabilize NPs. One of the main challenges is the incomplete knowledge regarding mechanistic aspects of the fungal production of NPs. Few studies already reported on molecules and moieties essential for the synthesis of NPs or their capping. However, the whole process of synthesis via biochemical pathways is often understudied. Understanding the precise roles of the biomolecules operating at the different steps of NP synthesis will provide us with techniques to manipulate the crystallinity, shape, size, dispersity, and other properties of the NPs.

In biomedical applications, it is important to understand how the biologically active compounds attach to the surfaces of NPs. This can increase their colloidal stability and biocompatibility, and it can provide additional properties, where the bioactive compounds are molecules with specific medical attributes. Large-scale cultivation methods for fungal NP synthesis need to be further developed to fully utilize the potential seen in some lab-scale experiments. Large-volume cultivation that creates relatively high yields of monodisperse NPs needs to be developed via synthesis processes close to standard temperature and pressure and with the use of fungal molecules without other reducing agents. This will make the production both cost effective and energy sustainable with a lower impact on the environment. Detailed study of the bioavailability of NPs and NP transport within tissues of the targeted organisms needs greater attention in the future, as this will allow their properties to be adjusted such that positive effects can be efficiently provided. When studies increase our knowledge in these critical areas, we will be close to the more extensive commercialization and use of tailor-made, highly effective, and environmentally benign mycosynthesized NPs.

## Figures and Tables

**Table 2:** Selected examples of ascomycetes used in the synthesis of various metal-containing NPs.

Species of Fungus	NP Type	Average Size (nm)	Synthesis Used	Additional Process Used	Source
*Aspergillus niger*	Ag	20	extracellular fungal culture		[33]
	Fe Fe_3_O_4_	18 50	fungal homogenate solution	supercritical condition of ethanol	[35]
*Aspergillus terreus*	Co_3_O_4_ CuO Fe_3_O_4_ NiO ZnO	10 19 32 43 30	cell-free filtrates	-	[52]
*Beauveria* spp.	Ag	25	extracellular fungal culture	-	[50]
*Fusarium oxysporum*	MnS NiS PbS ZnS	variable polydispersed fractal type structure	cell-free filtrates	-	[53]
*Humicola* sp.	Ag_2_S	20	cell-free filtrates	-	[54]
*Penicillium chrysogenum*	Ag	19 to 60	cell-free filtrates	-	[55]
	MgO	10	fungal melanin pigment	irradiation with gamma rays	[56]
	CuO	10	cell-free filtrates	irradiation with gamma rays	[57]
	ZnO	54	cell-free filtrates	-	[18]
*Phoma* sp.	Au	10 to 100	fresh biomass	-	[50]
*Trichoderma viride*	Ag	2 to 4	cell-free filtrates	-	[58]
	Au	20 to 30	cell-free filtrates	-	[59]

**Table 3:** Selected examples of basidiomycetes used in the synthesis of various metal-containing NPs.

Species of Fungus	NP Type	Average Size (nm)	Synthesis Used	Additional Process Used	Source
*Agaricus bisporus*	Ag	44	Fresh caps aqueous extract	-	[143]
	Pd	13	Aqueous extract	-	[144]
*Coprinus comatus*	Au	38 to 59	Fruit body aqueous extract	Ultraviolet irradiation 1 to 3 h	[145]
*Flammulina velutipes*	Au	74	Fresh fruit body aqueous extract	An additional catalyst to decrease color	[139]
*Ganoderma applanatum*	Ag	59	Fruiting body aqueous extract	-	[146]
*Ganoderma lucidum*	ZnO	30 to 50	Alcohol extract	-	[147]
	Ag Au	3 to 70 20 to 75	Intra- and extracellular aqueous extracts	-	[142]
*Grifola frondosa*	Ag Au	3 to 70 10 to 55	Intra- and extracellular aqueous extracts	-	[142]
*Lentinus edodes*	Ag Au	3 to 70 10 to 75	Intra- and extracellular aqueous extracts	-	[142]
*Pleurotus djamor*	TiO_2_	31	Fresh fruit body aqueous extract	-	[148]
*Pleurotus florida*	Ag	10	Fruit body aqueous extract	Microwave, visible, and UV irradiation	[21]
*Pleurotus ostreatus*	Ag Au	3 to 70 2 to 75	Intra- and extracellular aqueous extracts	-	[142]
*Pleurotus sajo-caju*	Ag Ag	23 37	Aqueous extract	-	[138]
